# 细胞转录组学和代谢组学整合策略表征突变影响谷氨酰胺合成酶酶活的关键代谢通路

**DOI:** 10.3724/SP.J.1123.2024.04003

**Published:** 2025-03-08

**Authors:** Ting LING, Jing SHI, Tingze FENG, Shaojun PEI, Siyi LI, Hailong PIAO

**Affiliations:** 1.中国科学院大连化学物理研究所, 中国科学院分离分析重点实验室, 辽宁 大连 116023; 1. CAS Key Laboratory of Separation Science for Analytical Chemistry, Dalian Institute of Chemical Physics, Chinese Academy of Sciences, Dalian 116023, China; 2.中国医科大学附属肿瘤医院胸外科肿瘤研究所, 辽宁省肿瘤医院暨研究所, 辽宁 沈阳 110042; 2. Cancer Research Institute, Department of Thoracic Surgery, Cancer Hospital of China Medical University, Liaoning Cancer Hospital & Institute, Shenyang 110042, China; 3.中国医科大学生命科学学院生物化学和分子生物学教研室, 辽宁 沈阳 110122; 3. Department of Biochemistry & Molecular Biology, School of Life Sciences, China Medical University, Shenyang 110122, China; 4.中国科学院大学, 北京 100049; 4. University of Chinese Academy of Sciences, Beijing 100049, China

**Keywords:** 谷氨酰胺合成酶突变, 转录组学, 代谢组学, 肺癌, 谷氨酰胺, glutamine synthetase mutation, transcriptomics, metabolomics, lung cancer, glutamine

## Abstract

谷氨酰胺合成酶(GS)是细胞中唯一可以从头合成谷氨酰胺的酶,在癌症代谢中扮演着重要角色。GS酶活缺陷突变往往导致严重的代谢疾病甚至是死亡。理解GS酶活降低对生理功能的影响可能为靶向治疗提供新的方向,同时对其内在机制的探索可以为治疗干预开辟新的途径。已报道的GS酶活缺陷突变包括R324C(第324位精氨酸突变为半胱氨酸)和R341C(第341位精氨酸突变为半胱氨酸)等,本研究新发现了GS的另一个酶活缺陷突变位点K241(第241位赖氨酸)。为了探究GS酶活缺陷突变带来的影响,本研究利用双组学技术对GS的酶活缺陷突变R324C和K241R(第241位赖氨酸突变为精氨酸)在肺癌细胞中的功能进行研究,通过对转录组和代谢组数据的整合揭示了GS酶活缺陷突变对一些重要生物过程的显著影响。GS的缺陷突变阻碍了细胞周期和多种氨基酸代谢途径。除了谷氨酰胺合成受到抑制,精氨酸-脯氨酸代谢、甘氨酸-丝氨酸-酪氨酸代谢以及天冬氨酸-谷氨酸-丙氨酸代谢在GS突变的细胞中表现出更强的活力,同时,氨酰-tRNA的生物合成途径也显著被激活。进一步的研究发现,GS酶活缺陷带来氨基酸代谢的改变源自谷氨酸的重新定向和相关代谢酶的表达变化,同时,氨酰-tRNA生物合成的激活源自谷氨酰胺在GS酶活缺陷细胞中带来的能量应激激活了特定蛋白的表达,如转录因子4(ATF4)。此外,细胞表型实验表明,GS酶活缺陷细胞的迁移能力低于野生型细胞。以上结果揭示了GS酶活缺陷突变体细胞中的代谢重编程现象,突出了癌细胞代谢的复杂性和适应性。

## Abstract (en)

Glutamine synthetase (GS), the only enzyme responsible for de novo glutamine synthesis, plays a significant role in cancer progression. As an example of the consequences of GS mutations, the R324C variant causes congenital glutamine deficiency, which results in brain abnormalities and neonatal death. However, the influence of GS-deficient mutations on cancer cells remains relatively unexplored. In this study, we investigated the effects of GS and GS-deficient mutations, including R324C and previously unreported K241R, which serve as models for GS inactivation. This study provided intriguing insights into the intricate relationship between GS mutations and cancer cell metabolism.

Our findings strongly support recent studies that suggest GS deletion leads to the suppression of diverse signaling cascades associated with glutamine metabolism under glutamine-stripping conditions. The affected processes include DNA synthesis, the citric acid cycle, and reactive oxygen species (ROS) detoxification. This suppression originates from the inherent inability of cells to autonomously synthesize glutamine under glutamine-depleted conditions. As a key source of reduced nitrogen, glutamine is crucial for the formation of purine and pyrimidine bases, which are essential building blocks for DNA synthesis. Furthermore, the citric acid cycle is inhibited by the absence of negatively charged glutamate within the mitochondrial matrix, particularly when glutamine is scarce. This deficiency decreases the flux of *α*-ketoglutarate (*α*-KG), a principal driver of the citric acid cycle. Intermediate metabolites of the citric acid cycle directly or indirectly contribute to the generation of nicotinamide adenine dinucleotide phosphate (NADPH) oxidase, a core component of redox homeostasis.

Using the GS_R324C and GS_K241R mutants, we conducted an integrative transcriptomics and metabolomics analysis. The GS mutants with reduced activity activated multiple amino acid biosynthesis pathways, including arginine-proline, glycine-serine-threonine, and alanine-aspartate-glutamate metabolism. This intriguing behavior led us to hypothesize that despite hindrance of the citric acid cycle, abundant intracellular glutamate is redirected through alternative processes, including transamination. Simultaneously, key metabolic enzymes in the amino acid synthesis pathways, such as glutamic-oxaloacetic transaminase 1 (GOT1), glutamic-pyruvic transaminase 2 (GPT2), pyrroline-5-carboxylate reductase 1 (PYCR1), and phosphoserine aminotransferase 1 (PSAT1), exhibited increased mRNA levels. Additionally, GS deficiency appeared to upregulate the expression of glutamine transporters SLC38A2 and SLC1A5. Thus, restricting extracellular amino acids, such as glutamine, induces a stress response while promoting transcription or translation by a select group of genes, thereby facilitating cellular adaptation. However, similar to GS_WT, both GS_R324C and GS_K241R were modulated by glutamine treatment.

Among GS-activity-dependent behaviors, the increased expression of numerous aminoacyl-tRNA synthetases (ARSs), which are critical for aminoacyl-tRNA biosynthesis, remains poorly understood. Most ARS-encoding genes are transcriptionally induced by activating transcription factor 4 (ATF4), the expression of which increases under oxidative stress, endoplasmic reticulum stress, hypoxia, and amino acid limitation. In GS-deficient cells, the increased expression of ATF4 was accompanied by pronounced stress caused by glutamine starvation. Thus, ARS upregulation may predominantly arise from increased ATF4 expression in GS-deficient cells. Additionally, transcriptomic analysis revealed the differential expression of specific genes, regardless of GS activity, suggesting that GS is involved in various processes other than glutamine synthesis, including angiogenesis. Although our omics study was limited to H1299 cells, in subsequent experiments, we validated our findings using additional cell lines, including Hepa1-6 and LN-229. To attain a more comprehensive understanding of the impact of the newly identified GS_K241R mutant, our investigation should be extended to various cell types and mouse models.

In summary, we identified and investigated GS-deficient mutations in cancer cells and conducted an integrative transcriptomics-metabolomics analysis with comparisons to wild-type GS. This comprehensive approach provided crucial insights into the intricate pathways modulated by GS activity. Our findings advance the understanding of how GS functions in the context of reprogrammed cellular metabolism, particularly during glutamine deprivation. The altered metabolism triggered by elevated glutamate levels arising from GS mutations highlights the remarkable plasticity of cancer cell metabolism. Notably, considering the increasing research focus on GS as a potential therapeutic target in various cancer types, the findings of this study could provide innovative perspectives for drug development and the formulation of clinical treatment strategies.

谷氨酰胺稳态的维持关系到细胞生理过程的正常进行。大多数癌症细胞的增殖显著地表现出谷氨酰胺成瘾的特征,最近的研究发现不同癌症类型或亚型的代谢过程均发生变化,这进一步印证了癌症是一种代谢疾病这一概念^[1,2]^。我们对癌症细胞代谢认知的进步已经改变了肿瘤有氧糖酵解的代谢特征仅仅源于线粒体功能障碍的观念。此外,人们认识到谷氨酰胺是一种多功能生物合成底物,可以通过生成*α*-酮戊二酸和柠檬酸参与氧化磷酸化和脂肪酸从头合成途径^[3]^。鉴于其多能性,谷氨酰胺在促进癌细胞的生长和存活方面发挥着关键作用^[4]^。

除谷氨酰胺外,其他氨基酸也可作为支持三羧酸(TCA)循环的替代能量库^[5,6]^。此外,氨基酸在嘌呤和嘧啶(核苷酸结构的重要组成部分)的合成中起着不可或缺的作用^[7⇓-9]^。例如丝氨酸在转化成甘氨酸的过程中提供了核苷酸代谢必需的一碳单位^[7]^。氨基酸衍生物也积极参与表观遗传调控和翻译后修饰^[10,11]^,例如氨基酸衍生的乙酰辅酶A是蛋白质乙酰化修饰的直接参与者^[12]^。此外,谷氨酸、甘氨酸和半胱氨酸直接参与谷胱甘肽的合成,同时丝氨酸驱动的叶酸循环是磷酸戊糖途径外细胞产生的还原型烟酰胺腺嘌呤二核苷酸磷酸(NADPH)的另一条主要途径,而谷胱甘肽和NADPH对于维持氧化还原稳态至关重要^[13,14]^。

细胞内的谷氨酰胺可从血液中获得或者通过谷氨酰胺合成酶(GS)催化的从头合成来产生。在肝脏和肾脏中,高表达的GS利用细胞内和细胞外的游离氨来调节酸碱平衡并消除高氨带来的氨中毒现象^[15,16]^。在神经系统中,尤其是星形胶质细胞中,GS承担着维持适当谷氨酸水平的责任,从而保护神经元免受兴奋性毒性的影响^[17]^。多种癌症中GS表达都显著升高,包括肝癌、乳腺癌、卵巢癌、胰腺癌和肺癌^[16,18⇓⇓⇓-22]^。谷氨酰胺是核苷酸产生的重要原料,肿瘤中这种增强的GS表达促进了谷氨酰胺的合成,进而促进了肿瘤的进展^[23]^。此外,新兴的研究强调了在特定癌症治疗中GS表达与耐药性之间的相关性,例如在吉非替尼耐药细胞中GS水平降低,而吉非替尼敏感的细胞中GS水平升高,这种差异表明GS在影响癌症耐药性和治疗结果方面具有潜在作用^[21,24]^。

尽管如此,对突变引起GS酶活缺陷的报道相对较少,仅在患者病例中报告了少数突变,包括R324C和R341C^[25,26]^。此外,对于GS酶活缺陷所产生的生物学效应和生理功能也罕有报道。本研究通过全面整合转录组学和代谢组学分析弥补空缺,目的是揭示GS酶活缺陷突变体R324C和K241R(第241位赖氨酸突变为精氨酸)对肺癌细胞代谢途径的调节和影响,研究结果最终揭示了GS酶活缺陷突变对氨基酸代谢相关的特定信号通路产生了显著的影响。

## 1 实验部分

### 1.1 仪器与试剂

G7100A毛细管电泳仪、G6224A飞行时间质谱仪(TOF-MS)和8890-G3542A气相色谱-质谱仪(GC-MS)购自美国Agilent公司。毛细管电泳-质谱(CE-MS)代谢分析中使用的熔融石英毛细管(80 cm×50 μm i.d.)购自日本HMT公司。用来进行定量PCR反应的CFX-96仪购自美国Bio-Rad公司。

人肺癌细胞系H1299购自中国科学院典型培养物保藏委员会细胞库(CTCC)。人胚肾细胞系HEK293T、人胶质瘤细胞系LN-229和小鼠肝癌细胞系Hepa1-6来自美国典型培养物保藏中心(ATCC)。完全或不含谷氨酰胺的RPMI-1640细胞培养基、完全或不含谷氨酰胺的DMEM细胞培养基购自中国美仑生物科技有限公司。细胞培养所用血清(FBS)购自美国GBIOC公司,青霉素/链霉素、CO_2_细胞加湿培养箱购自美国Thermo公司。蛋白酶和磷酸酶抑制剂购自美国Bimake公司。RNA提取所用的RNAiso Plus试剂、反转录试剂PrimeScript^TM^ RT Master Mix均购自日本TaKaRa公司。实时荧光定量PCR所用试剂StepOnePlus和SYBR-Green Ⅰ均购自瑞士Roche公司。磷酸盐溶液(PBS)、引物购自上海生工生物工程公司。

抗体:抗GS一抗购自美国BD Biosciences公司,抗Vinculin抗体、抗S6抗体购自美国Santa Cruz公司,抗pS6抗体购自美国Cell Signaling公司。慢病毒载体PBOBI载体和LentiCRISPR-V2均来自厦门大学林圣彩实验室。

### 1.2 细胞培养、质粒构建与细胞侵染

细胞培养^[27]^: H1299细胞接种在含有10%FBS的RPIM-1640完全培养基中培养。Hepa1-6、HEK293T和LN-229细胞均接种在含有10%FBS的DMEM完全培养基中培养。在制备样品时,用PBS洗涤细胞并在不含谷氨酰胺的培养基中培养。

质粒构建^[28]^:将重叠延伸PCR产生GS的突变体克隆到PBOBI载体中。所有质粒的验证均通过DNA测序的方法进行。GS敲除的慢病毒载体的构建:将单导向*GLUL* (sg*GLUL*)序列克隆到LentiCRISPR-V2载体中,具体来说,利用单酶切连接的方法将退火的单导向RNA(sgRNA)序列连接到位于线性化载体U6启动子后的BsmbI位点上。靶向*GLUL*的序列为Human *GLUL* sgRNA (AAGTACAATCGAAGGCCTGC)。

细胞侵染^[28]^:转染48 h后收集含有病毒的上清液,通过0.45 μm滤膜过滤去除细胞碎片,然后添加到靶细胞培养基中。18 h后,用对应的抗生素进行筛选。

### 1.3 谷氨酰胺合成酶活性检测

GS催化的*γ*-谷氨酰胺羟肟酸转移实验按照Haghighat^[29]^的描述进行。简而言之,用150 μL 50 mmol/L咪唑水溶液(pH 6.8)重悬细胞后,将其置于-80 ℃冰箱中,30 min后取出样品并置于室温进行解冻,随后离心,取上清,获得细胞裂解液。取50 μL细胞裂解液,用于蛋白质印迹定量,剩余裂解液与等体积的反应缓冲液(含50 mmol/L咪唑、20 mmol/L Na_2_HPO_4_、0.16 mmol/L二磷酸腺苷(ADP)、50 mmol/L谷氨酰胺(Gln)、25 mmol/L羟胺、2 mmol/L MnCl_2_的水溶液)混合并在37 ℃下孵育45 min。用400 μL终止液(含2.42% FeCl_3_、1.45%三氯乙酸和1.82%HCl的水溶液)终止反应。在540 nm处测定吸光度。

### 1.4 RNA提取和实时荧光定量PCR(qPCR)

将细胞接种于6 cm培养皿中,并在含有10%血清的DMEM或RPIM-1640培养基中培养。对于样品制备,待细胞完全贴壁后用PBS洗涤细胞并在无谷氨酰胺的培养基中培养8 h。如文献^[28]^所述,通过RNAiso Plus试剂进行细胞样品的RNA抽提,使用PrimeScript^TM^ RT Master Mix通过逆转录反应获得cDNA。实时荧光定量PCR由StepOnePlus和用于检测的DNA双链特异性试剂SYBR-Green Ⅰ在CFX-96仪器中进行。引物序列如表1所示。

**表1:** qPCR所用的引物信息

Gene	Forward sequence	Reverse sequence
GLUL	GCCTCAGGGTGAGAAAGTCC	TCCGTTTACAGGTGTGCCTC
GLNS2	GGCACATGCCAAGCTGGATA	AGTGGCTCTGCTAGGGAGAA
POLE2	TGTTTGTACCTGGTCCAGAGG	TCTTGGAAAAGAGCCAGGGT
FEN1	CCCAAAGGCCAGTCATCCCT	GCGGTAGAACATGCCCATCA
MCM4	TCCTTGTCGCGCAGGTACT	TCAAAGTCAAGAGGGATAGCTGA
CCNB1	GACCTGTGTCAGGCTTTCTCTG	GGTATTTTGGTCTGACTGCTTGC
CCND1	TCTACACCGACAACTCCATCCG	TCTGGCATTTTGGAGAGGAAGTG
CCND3	AGATCAAGCCGCACATGCGGAA	ACGCAAGACAGGTAGCGATCCA
CDC6	GGAGATGTTCGCAAAGCACTGG	GGAATCAGAGGCTCAGAAGGTG
ATF4	TTCTCCAGCGACAAGGCTAAGG	CTCCAACATCCAATCTGTCCCG

### 1.5 基于CE-MS的代谢组学分析

使用甲醇(700 μL)进行细胞刮取,使用氯仿(700 μL)对样品进行涡旋后加入超纯水(280 μL)涡旋并静置5 min,在4 ℃下以15000 g离心15 min,并将过滤的水相冷冻干燥。随后,样品通过熔融石英毛细管进行色谱分离,使用CE系统结合TOF-MS进行整体代谢物分析。CE-MS分析方法按照先前建立的步骤^[30]^进行。代谢组学数据根据总峰面积矫正标准化后,使用MeV 4.8.1进行热图可视化。采用SIMCA软件进行PLS-DA分析,对投影中的可变重要性(VIP)值大于1的代谢物进行单因素方差分析从而开展多组间比较。通过基于网络的MetaboAnalyst(https://genap.metaboanalyst.ca/MetaboAnalyst/)进行差异蛋白通路富集分析。

### 1.6 基于GC-MS的代谢分析

对于GC-MS样品的准备,提前对接种在10 cm培养皿中的细胞进行8 h谷氨酰胺饥饿处理,将培养基更换为含0.2 mmol/L谷氨酰胺、2 mmol/L ^15^NH_4_Cl的RPIM-1640培养基。标记0.5 h后,用0.9% NaCl洗涤细胞,并用液氮淬灭,以进行下一步代谢物提取。使用80%甲醇水溶液进行细胞中代谢物的提取,在4 ℃下以15000 g离心15 min,取上清液进行冷冻干燥。代谢物衍生化按照先前建立的步骤进行^[31]^。样品在DB-624 MS毛细管柱(20 m×0.18 mm×1 μm, Agilent Technologies,美国)上分离,在70 eV电子轰击下运行的单四极杆质谱仪中电离;进样口、接口和离子源温度均保持在230 ℃,四极杆温度保持在150 ℃;以1∶21的分流比进样1 μL样品;载气氦气流速恒定为0.95 mL/min,柱温起始于35 ℃,并以25 ℃/min的速率升高直至240 ℃;在选择离子监测(SIM)模式下进行代谢物同位素谱分析。

### 1.7 RNA测序和生物信息学分析

RNA测序由广州锐博生物科技有限公司完成,测序的具体过程见文献[32]。简而言之,对总RNA进行清洁和富集后,进行逆转录反应,并在反应中加入barcode和适配子序列,制备的cDNA库经过质量评估并使用Illumina平台(Illumina,美国)测序。采用DESeq2软件进行基因差异表达分析。调整后的*P*值小于0.05且log_2_ Fold change大于1的基因被归类为正差异表达基因。使用R编程语言进行通路富集分析。

### 1.8 Transwell迁移实验

Transwell迁移实验按照文献[33]所述进行。将约3×10^5^个细胞接种在带有未包被膜(24-well insert, BD Biosciences,美国)的上室中,室中加入200 μL不含血清和谷氨酰胺的RPMI-1640培养基。将700 μL不含谷氨酰胺的RPMI-1640培养基(含20% FBS)倒入下室中。Transwell板在37 ℃下孵育10 h。将通过孔迁移的细胞用冷甲醇固定,用结晶紫染色,并在光学显微镜下计数,以确定每个实验组中迁移细胞的数量。

### 1.9 统计分析

每个实验重复3次或以上。除非另有说明,数据表示为平均值±标准误差。使用*t*检验(不配对,双尾)进行分析,以比较两个独立样本的差异。通过*t*检验分析的数据服从正态分布,我们进行*F*检验来比较方差,结果没有显著差异。因此,当使用未配对*t*检验时,我们假设方差相等,并且没有样本被排除在分析之外。*P*<0.05表示有统计学意义。

## 2 结果与讨论

### 2.1 GS酶活关键位点K241的鉴定

在之前关于GS的苏木化的研究^[34]^中,我们发现第241位赖氨酸(K241)的突变对GS的酶活产生了重大影响。为了更加全面地了解K241R突变体的酶活,我们在HEK293T细胞中过表达不同剂量的K241R突变体和野生型GS质粒,并对细胞进行了体外酶活检测。值得注意的是,与野生型GS相比,K241R突变体的酶活性明显降低(图1a)。为了排除精氨酸引入带来的非特异性影响,我们在K241处引入不同氨基酸(包括丙氨酸和甘氨酸)突变。正如预期的那样,在突变成其他氨基酸后,K241位的GS突变体依然表现出低于野生型蛋白的酶活,这表明K241这个位点在协调谷氨酰胺合成活性中可能具有关键作用(图1b)。此外,与已被报道的GS酶活缺陷突变R324C相比,K241R突变GS显示出相似的酶活水平(图1c)。

**图1:**
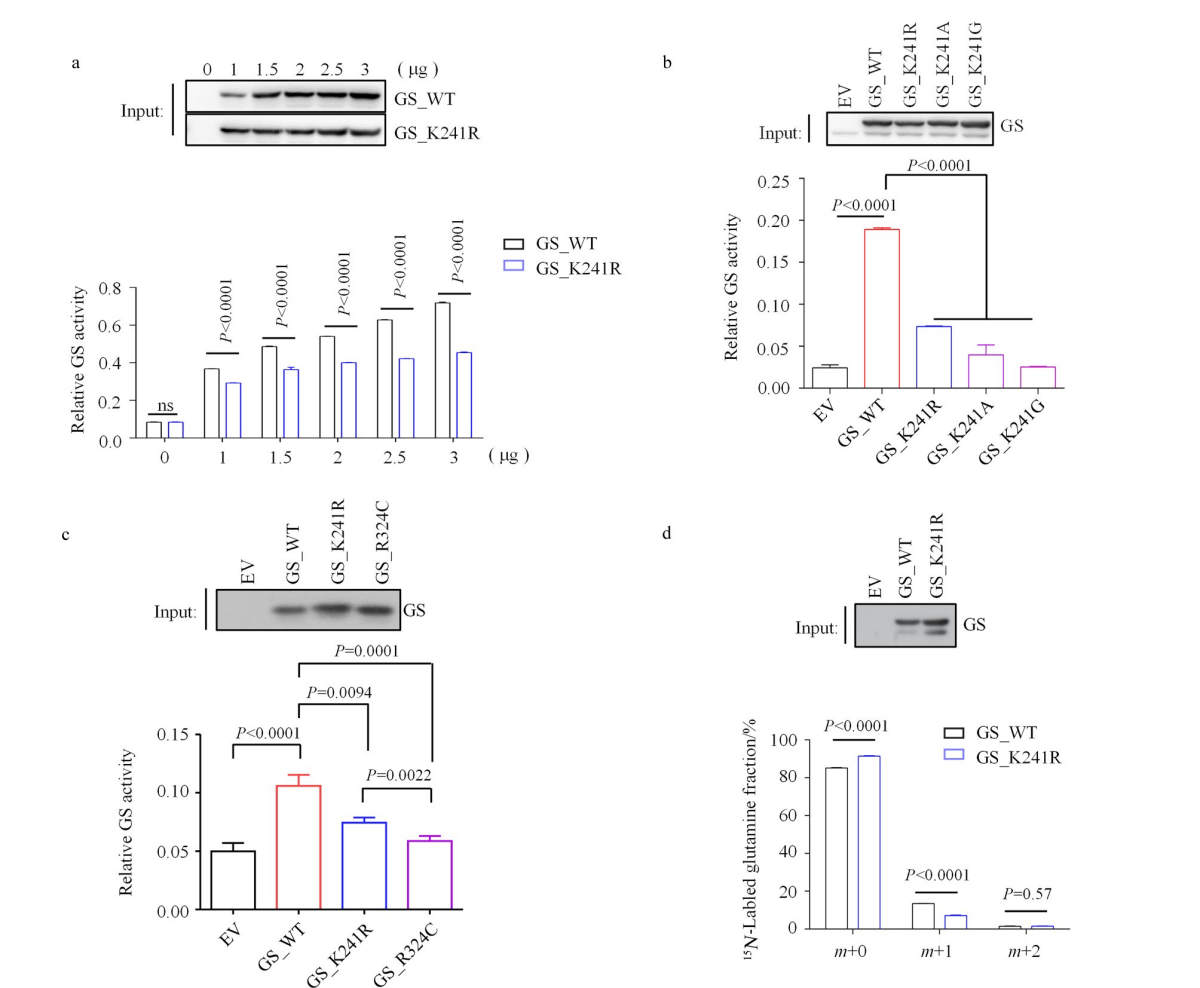
GS和突变体K241R的酶活

为了进一步确认GS酶活缺陷突变体K241R的酶活水平,我们在已经敲除GS的肺癌细胞系H1299中重新进行了GS野生型、突变体K241R以及空载体(EV)的引入。单胺氧化酶A(MAOA)是调节生物体内氨的最重要的酶之一^[35]^,而以上细胞系中MAOA的mRNA水平并无二致(附图1a,详见www.chrom-China.com),这说明细胞内氨的产生不存在明显差异。之后,对表达GS野生型或突变体K241R的细胞在含^15^N标记的氯化铵、不含谷氨酰胺的RPMI-1640培养基中进行了30 min的标记后进行GC-MS分析。结果显示,在GS野生型细胞中,大约13%的谷氨酰胺被标记,而K241R细胞仅仅只有6%的标记量(图1d)。这种显著的差异再一次验证了GS的K241处的突变是酶活缺陷突变。

### 2.2 通过RNA测序分析GS调控的转录本

为了进一步探究GS调控的基因表达,我们对GS敲除后的H1299细胞进行了RNA测序分析(图2a)。

**图2:**
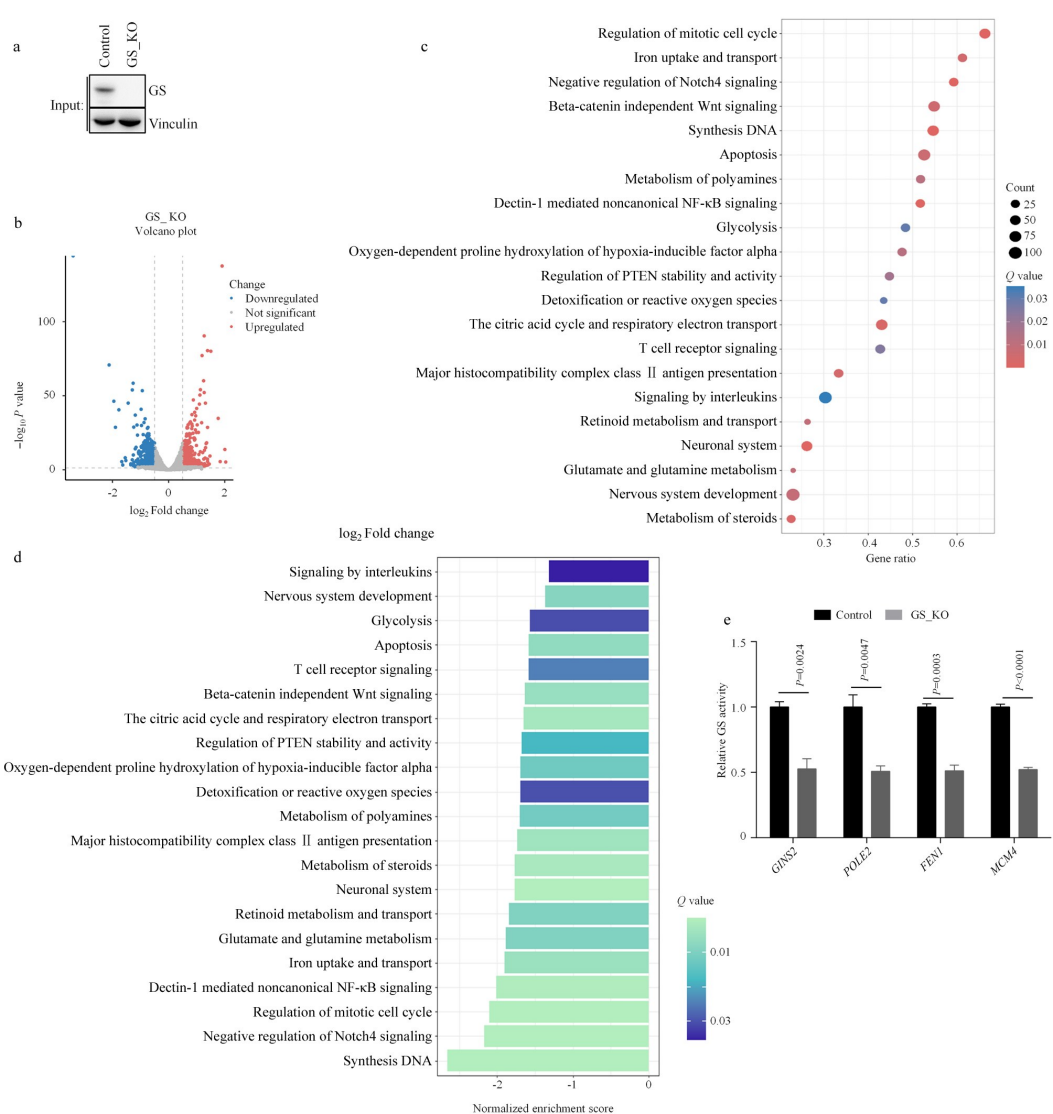
GS敲除细胞的RNA测序分析

与对照组细胞相比,GS敲除组细胞中183个基因上调,218个基因下调(图2b)。使用京都基因与基因组百科全书(KEGG)数据库进行进一步分析得到了与变化基因相关的生物过程,包括细胞有丝分裂、DNA合成和细胞凋亡等(图2c)。

随后,采用标准化富集评分分析发现了一些信号通路在GS敲除的细胞中表现出明显的抑制状态,包括与离子的摄取与转运、神经系统发育和糖酵解信号传导相关的途径等(图2d)。此外,我们在H1299中重新进行了RNA的提取,并对DNA合成相关的基因的转录水平进行了验证,包括*GLNS2*、*POLE2*、*FEN1*和*MCM6*,结果显示这些基因的转录在GS酶活缺陷的细胞系中受到抑制(图2e)。为了验证结果的普适性,在GS敲除的人胶质细胞LN-229和小鼠肝细胞Hepa1-6中,我们对上述DNA合成相关的基因同样进行了验证,得到了相同的结论(附图2a)。

### 2.3 筛选依赖GS酶活性的下游信号途径

高表达的GS通过促进谷氨酰胺合成显著促进肿瘤进展。尽管人们不断探索潜在的分子机制,但对GS酶活缺陷引起的转录变化仍然了解有限^[36]^。为了探究GS酶活缺陷对细胞的影响,在GS敲除后的H1299细胞中重新引入了GS野生型以及新发现的酶活缺陷突变体K241R和前面提到的新生儿致死突变R324C(图3a)。随后,通过RNA测序,揭示了不同组间差异表达的基因(图3b)。此外,通过比较GS野生型和不同突变体之间的基因,分别在表达GS_K241R和GS_R324C的细胞中鉴定了1462个和1158个差异基因(图3c)。KEGG分析进一步证实了GS酶活缺陷对氨基酸生物合成、DNA复制和细胞周期相关的基因的表达产生了巨大影响(仅举几例,如图3d所示)。此外,调节细胞周期的特定基因(如*CCNB1*、*CCND1/3*和*CDC6*),在表达GS酶活缺陷突变的H1299细胞中相比表达野生型GS的细胞表现出显著的下调(如图3e所示)。同时,我们在敲除GS的LN-229细胞中同样进行了GS野生型和突变体回补的稳定细胞系的构建(附图1c),并对上述细胞周期相关的基因转录水平进行了检测,结果与H1299细胞类似(附图2b)。

**图3:**
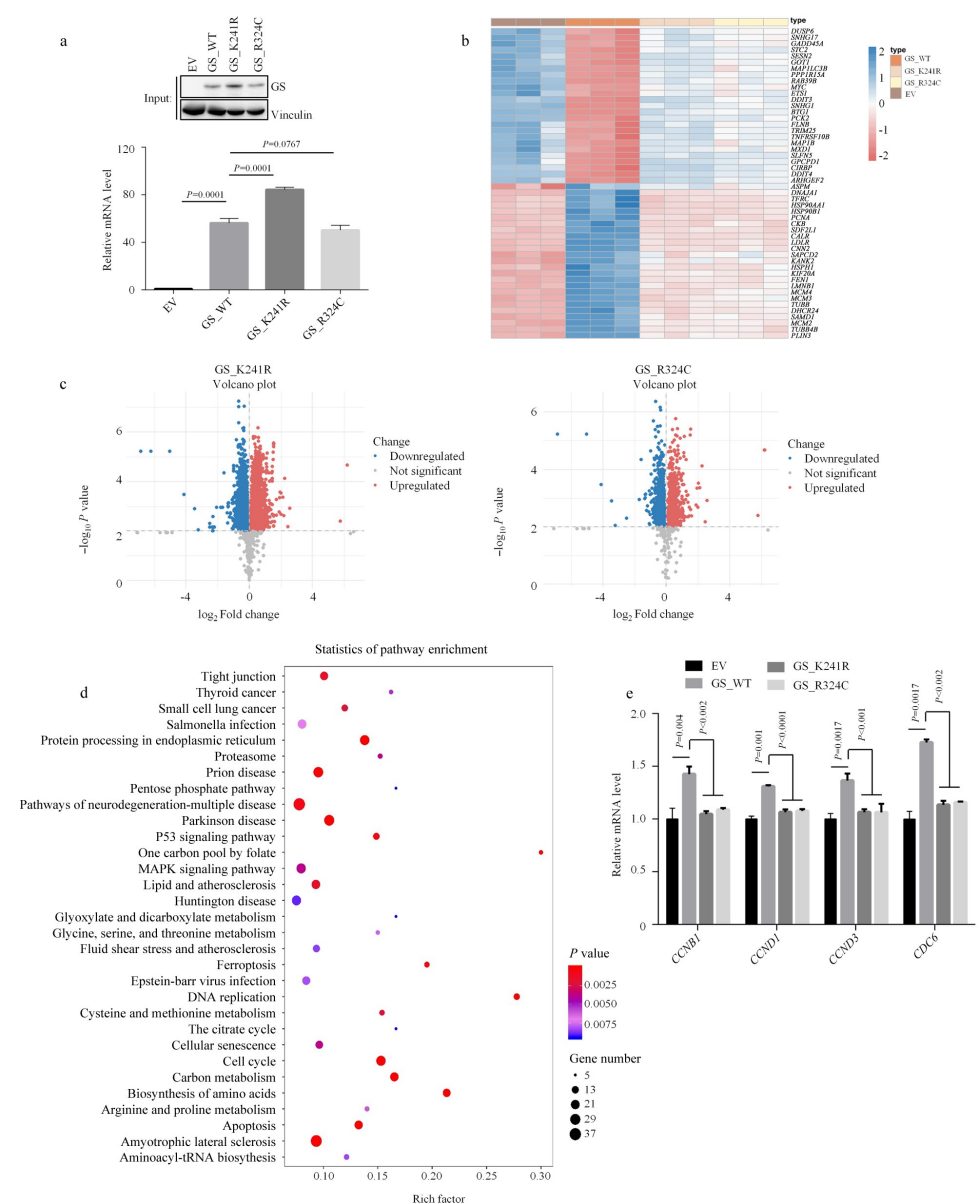
H1299细胞中GS重新敲入的RNA测序分析

### 2.4 确定GS酶活缺陷影响的代谢途径和代谢物

GS酶活缺陷可能导致新生儿早夭,同时GS的异常表达在多发性骨髓瘤和少突胶质细胞瘤中已得到证实^[26,37,38]^。为了揭示GS酶活缺陷突变引起的代谢变化,本研究利用CE-MS对上述提到的GS野生型和突变体稳转H1299细胞系进行了代谢组学分析,并对得到的代谢物进行了热图展示(附图3a)。PLS-DA分析发现,4个组别(EV、GS_WT、GS_K241R和GS_R324C)得到了很好的区分(附图3b, c)。利用MetaboAnalyst软件对主成分中VIP值大于1的代谢物(附图3d)进行代谢通路分析,结果显示精氨酸生物合成和嘌呤代谢等途径在野生型和突变GS组中存在显著差异(图4a)。此外,通过比较GS野生型和突变体细胞之间的代谢数据,我们得到了44种差异代谢物(图4b)。上述结果表明GS酶活缺陷可以影响包括精氨酸-脯氨酸在内的多种氨基酸代谢途径,我们对其中的一些代谢物的含量进行了详细表征(图4c)。

**图4:**
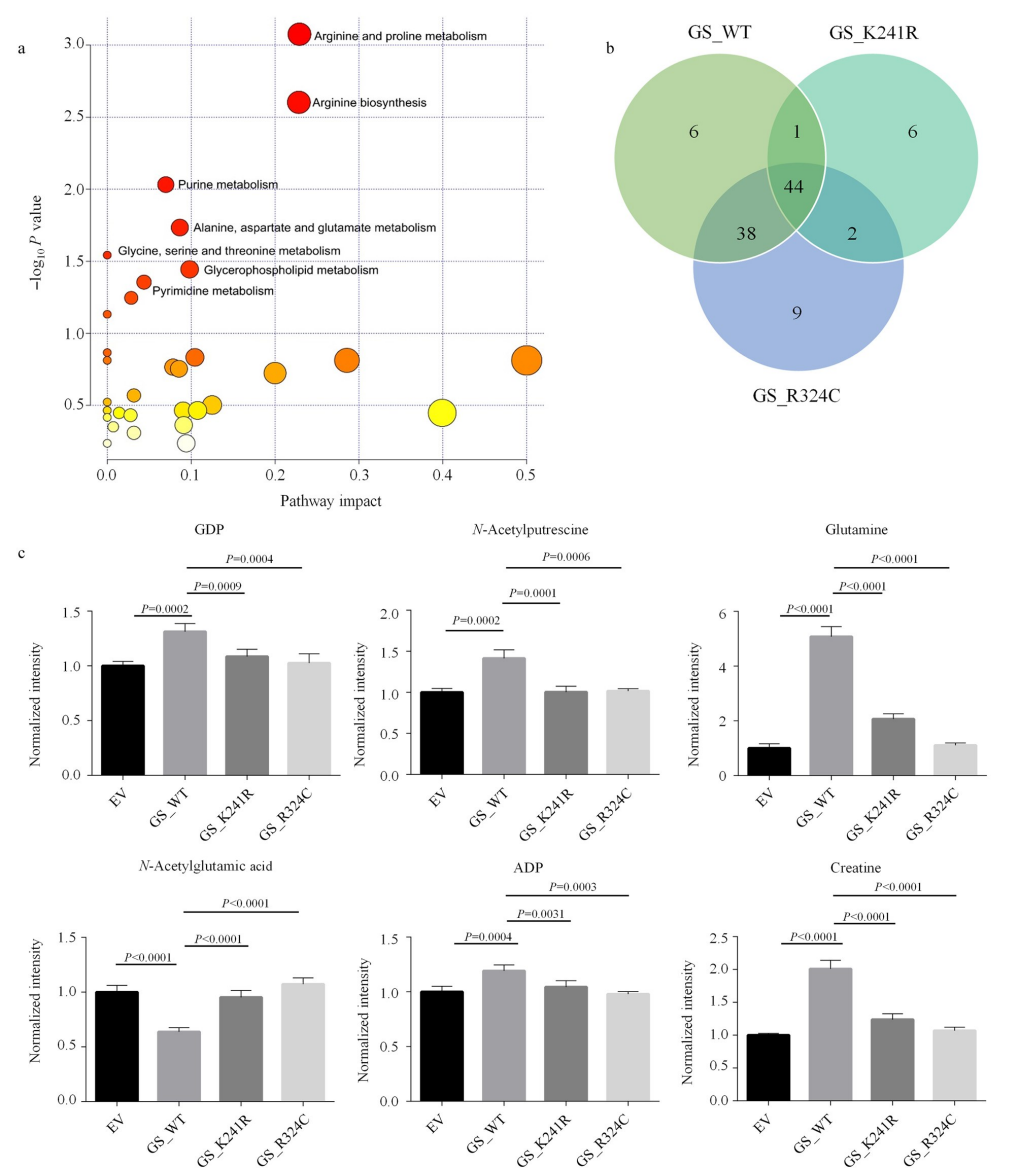
GS突变对H1299细胞中代谢物和代谢通路的影响

### 2.5 探索GS突变影响代谢途径的机制

生物过程本质上具有复杂性和整体性,通常需要采取多方面的手段来充分揭示疾病发生和进展的复杂机制。采用代谢组学分析和转录组学分析相结合的方法可以提供双重视角,从而更全面地了解GS酶活变化对生物过程的影响。双组学整合的研究结果揭示了响应GS酶活变化的4个途径,即精氨酸-脯氨酸代谢、甘氨酸-丝氨酸-苏氨酸代谢、天冬氨酸-谷氨酸代谢以及氨酰-tRNA生物合成(图5a)。

**图5:**
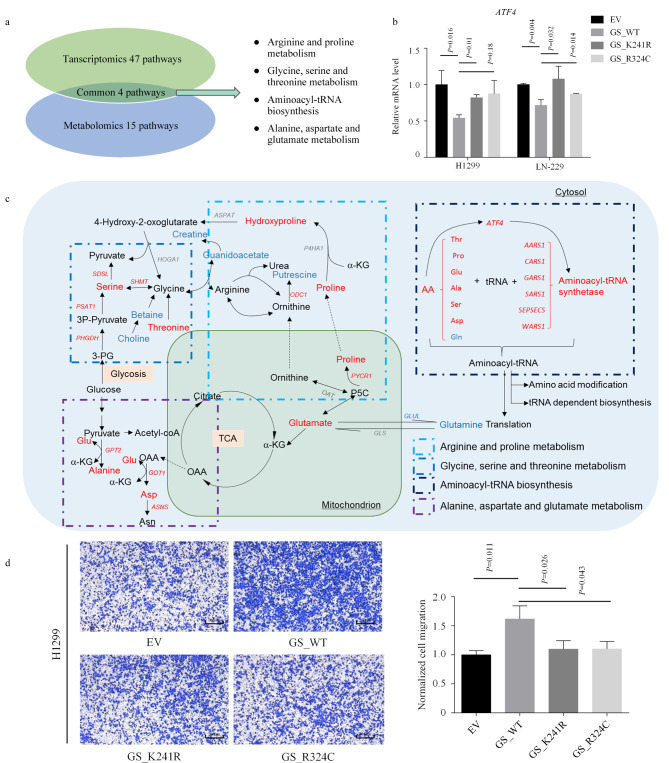
转录组学-代谢组学整合分析GS酶活缺陷突变影响的代谢通路

对氨基酸代谢途径的进一步整合揭示了其中关键的差异表达基因,包括与精氨酸-脯氨酸代谢相关的基因(*PYCR1*、*ODC1*、*SAT1*、*SMOX*),与甘氨酸-丝氨酸-苏氨酸代谢相关的基因(*DMGDH*、*PHGDH*、*PSAT1*、*SDSL*、*SHMT*)(附图4b),以及与天冬氨酸-谷氨酸-丙氨酸代谢相关的基因(*ASNS*、*GOT1*、*GPT2*)(附图4c)。我们在H1299和LN-229的GS稳转细胞系中重新对ASNS和GOT1的转录水平进行了检测,结果与RNA测序的结果吻合(附图2c)。在谷氨酰胺饥饿条件下GS酶活缺陷的细胞的ASNS和GOT1水平有所提高,这可能是由这两种酶各自的底物天冬酰胺和谷氨酸水平升高所引起的;同时,这种条件下GS酶活缺陷细胞多种氨基酸水平的增加和氨酰-tRNA合成酶(ARS)表达的增加(附图4d, e),说明尽管在GS酶活缺陷细胞中谷氨酰胺的合成受到抑制,但是细胞通过其他氨基酸的补充和ARS的上调进行弥补,维持RNA翻译并促进tRNA依赖性的生物修饰。同时,谷氨酰胺合成的减少激活了H1299和LN-229中转录因子4(ATF4)的表达(图5b)。ATF4是一种应激诱导的转录因子,负责大多数编码胞质ARS基因的转录^[39,40]^。概括说来,谷氨酰胺饥饿导致GS酶活缺陷细胞中的谷氨酰胺含量明显低于野生型细胞,而谷氨酰胺水平的降低引起了谷氨酸水平的升高和其他代谢物的变化,同时相关基因的表达也受到影响(图5c)。细胞迁移能力与谷氨酰胺合成能力息息相关,为揭示GS酶活缺陷的生理功能,我们对细胞的迁移能力进行了检测,结果显示表达GS野生型的细胞表现出更强的迁移能力,而表达GS酶活缺陷突变体K241R和R324C的细胞其迁移能力明显受到抑制(图5d)。

### 2.6 讨论

我们的研究结果从新的角度支持了最近的报道,即在谷氨酰胺剥离条件下,GS的缺失会导致与谷氨酰胺代谢相关的多种信号级联被抑制^[4,41,42]^。这些过程包括DNA合成、柠檬酸循环和活性氧(ROS)解毒等。这种抑制源于GS酶活缺陷细胞在谷氨酰胺耗尽的条件下细胞无法正常自主合成谷氨酰胺。

利用GS的R324C和K241R突变体,我们进行了转录组学和代谢组学的整合分析。这些酶活性降低的GS突变体激活了多种氨基酸生物合成途径,例如精氨酸和脯氨酸代谢,甘氨酸-丝氨酸-苏氨酸代谢和丙氨酸-天冬氨酸-谷氨酸代谢。此外,GS酶活缺陷似乎会导致谷氨酰胺转运蛋白SLC38A2和SLC1A5表达上调(附图2d)。事实上,细胞外氨基酸(例如谷氨酰胺)限制会引发应激反应,促使一组特定的基因转录或翻译上调,从而使细胞适应变化^[43⇓-45]^。此外,与野生型GS类似,R324C和K241R突变体在谷氨酰胺饥饿处理时蛋白水平都有所提高,这说明GS酶活水平的高低似乎并不会影响其受到谷氨酰胺浓度调控的这一特征(附图1b)。

在GS酶活依赖性数据整合分析中,令人困惑的方面在于许多ARS的表达增强,而ARS是氨酰基-tRNA生物合成的关键参与者。据报道,大多数ARS编码基因是由ATF4转录诱导的,在氧化应激、内质网应激和缺氧以及氨基酸限制的条件下,ATF4的表达增加^[46]^。考虑到GS酶活缺陷的细胞中ATF4表达增加,再加上这些细胞中谷氨酰胺饥饿会造成更明显的应激,有理由认为ARS的上调主要来自GS酶活缺陷背景下ATF4表达的增加。此外,转录组学数据分析发现,与对照细胞相比,表达野生型或酶活缺失的GS蛋白的某些基因都表现出差异表达,证实了GS在细胞中的多功能性,例如其在谷氨酰胺合成之外的血管生成中的作用^[31]^。我们的组学研究范围仅限于H1299细胞,但在随后的实验中,我们使用其他细胞系(包括Hepa1-6和LN-229细胞)验证了我们的发现,确认了本研究发现的可靠性。为了更全面地理解新发现的GS的K241R突变影响的信号途径和生理过程,后续我们会将研究范围扩展到更多细胞类型及小鼠模型中,为揭示GS的生物学功能提供更多线索。

## 3 结语

综上,我们发现了影响GS酶活的关键突变,并在肺癌细胞中通过转录组学和代谢组学对GS以及其酶活缺陷突变体影响的信号通路和代谢过程进行了对比与整合,为GS以酶活依赖方式调节的复杂途径提供了重要信息。这项研究扩展了我们对GS如何在细胞代谢重编程背景下,特别是在谷氨酰胺缺乏时发挥作用的理解。GS酶活缺陷引起的谷氨酸水平升高引发的代谢重编程提示了癌细胞代谢的高度可塑性。值得注意的是,考虑到GS作为各种癌症前瞻性治疗靶点的新兴研究不断涌现^[20,21,36⇓-38,47,48]^,该研究可能会对靶向GS酶活的治疗药物的开发提供创新性的临床治疗参考。

## References

[1] Gyamfi J, Kim J, Choi J. Int J Mol Sci, 2022, 23(3): 1155 35163079 10.3390/ijms23031155 PMC8835572

[2] Seyfried T N, Shelton L M. Nutr Metab, 2010, 7(1): 7 10.1186/1743-7075-7-7 PMC2845135 20181022

[3] Seyfried T N. Front Cell Dev Biol, 2015, 3: 43 26217661 10.3389/fcell.2015.00043 PMC4493566

[4] Yang L, Moss T, Mangala L S, et al. Mol Syst Biol, 2014, 10(5): 728 24799285 10.1002/msb.20134892 PMC4188042

[5] Green C R, Wallace M, Divakaruni A S, et al. Nat Chem Biol, 2016, 12(1): 15 26571352 10.1038/nchembio.1961 PMC4684771

[6] Hensley C T, Wasti A T, DeBerardinis R J. J Clin Invest, 2013, 123(9): 3678 23999442 10.1172/JCI69600 PMC3754270

[7] Locasale J W. Nat Rev Cancer, 2013, 13(8): 572 23822983 10.1038/nrc3557 PMC3806315

[8] Shuvalov O, Petukhov A, Daks A, et al. Oncotarget, 2017, 8(14): 23955 28177894 10.18632/oncotarget.15053 PMC5410357

[9] Zhang Y, Morar M, Ealick S E. Cell Mol Life Sci, 2008, 65(23): 3699 18712276 10.1007/s00018-008-8295-8 PMC2596281

[10] Lieu E L, Nguyen T, Rhyne S, et al. Exp Mol Med, 2020, 52(1): 15 31980738 10.1038/s12276-020-0375-3 PMC7000687

[11] Pegg A E. IUBMB Life, 2009, 61(9): 880 19603518 10.1002/iub.230 PMC2753421

[12] Pietrocola F, Galluzzi L, Bravo-San Pedro J M, et al. Cell Metab, 2015, 21(6): 805 26039447 10.1016/j.cmet.2015.05.014

[13] Fan J, Ye J, Kamphorst J J, et al. Nature, 2014, 510(7504): 298 24805240 10.1038/nature13236 PMC4104482

[14] Chung W J, Lyons S A, Nelson G M, et al. J Neurosci, 2005, 25(31): 7101 16079392 10.1523/JNEUROSCI.5258-04.2005 PMC2681064

[15] Janicki R H, Goldstein L. Am J Physiol, 1969, 216(5): 1107 5768059 10.1152/ajplegacy.1969.216.5.1107

[16] Wasfy R E, Shams Eldeen A A. Asian Pac J Cancer Prev, 2015, 16(11): 4769 26107238 10.7314/apjcp.2015.16.11.4769

[17] Suárez I, Bodega G, Fernández B. Neurochem Int, 2002, 41(2/3): 123 12020613 10.1016/s0197-0186(02)00033-5

[18] Strohecker A M. Cancer Discov, 2013, 3(11): 1272 23965987 10.1158/2159-8290.CD-13-0397 PMC3823822

[19] Annese T, Tamma R, Ruggieri S, et al. Cancers (Basel), 2019, 11(3): 381 30889903 10.3390/cancers11030381 PMC6468440

[20] Fan S, Wang Y, Zhang Z, et al. J Cell Biochem, 2018, 119(7): 6008 29575012 10.1002/jcb.26797

[21] Wang L, Peng W, Wu T, et al. Cell Death Discov, 2018, 4: 24 10.1038/s41420-018-0086-x PMC6085389 30109143

[22] Wang Y, Fan S, Lu J, et al. J Cell Biochem, 2017, 118(8): 2018 27791265 10.1002/jcb.25775

[23] Tardito S, Oudin A, Ahmed S U, et al. Nat Cell Biol, 2015, 17(12): 1556 26595383 10.1038/ncb3272 PMC4663685

[24] Vettore L, Westbrook R L, Tennant D A. Br J Cancer, 2020, 122(2): 150 31819187 10.1038/s41416-019-0620-5 PMC7052246

[25] Häberle J. NEJM, 2005, 353(18): 1926 16267323 10.1056/NEJMoa050456

[26] Häberle J, Shahbeck N, Ibrahim K, et al. Molecular Genetics and Metabolism, 2011, 103(1): 89 21353613 10.1016/j.ymgme.2011.02.001

[27] Yan M, Liu J, Xia T, et al. Chinese Journal of Chromatography, 2019, 37(8): 887 31642260 10.3724/SP.J.1123.2018.07008

[28] Xia T, Chen D, Liu X, et al. Cell Death & Disease, 2022, 13(4): 314 35393397 10.1038/s41419-022-04736-6 PMC8990078

[29] Haghighat N. Neurochem Res, 2005, 30(5): 661 16176070 10.1007/s11064-005-2754-5

[30] Zeng J, Yin P, Tan Y, et al. J Proteome Res, 2014, 13(7): 3420 24853826 10.1021/pr500390y

[31] Eelen G, Dubois C, Cantelmo A R, et al. Nature, 2018, 561(7721): 63 30158707 10.1038/s41586-018-0466-7

[32] Xu X, Wang X, Chen Q, et al. J Transl Med, 2023, 21(1): 307 37147632 10.1186/s12967-023-04141-3 PMC10163764

[33] Piao H L, Yuan Y, Wang M, et al. Nat Cell Biol, 2014, 16(3): 245 24509793 10.1038/ncb2909 PMC3943677

[34] Ling T, Li S, Chen H, et al. FASEB J, 2023, 37(12): e23319 38010918 10.1096/fj.202301462RR

[35] Hu S, Deng L, Wang H, et al. Cytotechnology, 2011, 63(3): 247 21298341 10.1007/s10616-011-9336-y PMC3081043

[36] MacKerell A, Frieg B, Görg B, et al. PLOS Comput Biol, 2016, 12(2): e1004693 26836257 10.1371/journal.pcbi.1004693 PMC4737493

[37] Bolzoni M, Chiu M, Accardi F, et al. Blood, 2016, 128(5): 667 27268090 10.1182/blood-2016-01-690743

[38] Chiu M, Taurino G, Bianchi M G, et al. Int J Mol Sci, 2018, 19(4): 1099 29642388 10.3390/ijms19041099 PMC5979401

[39] Shan J, Zhang F, Sharkey J, et al. Nucleic Acids Res, 2016, 44(20): 9719 27471030 10.1093/nar/gkw667 PMC5175342

[40] Sung Y, Yoon I, Han J M, et al. Exp Mol Med, 2022, 54(5): 553 35501376 10.1038/s12276-022-00765-5 PMC9166799

[41] Yang L. Annu Rev Biomed Eng, 2017, 19: 163 28301735 10.1146/annurev-bioeng-071516-044546

[42] Chen L, Cui H. Int J Mol Sci, 2015, 16(9): 22830 26402672 10.3390/ijms160922830 PMC4613338

[43] Stretton C, Lipina C, Hyde R, et al. Biochim Biophys Acta Mol Cell Res, 2019, 1866(6): 978 30857869 10.1016/j.bbamcr.2019.03.002 PMC6456927

[44] Gazzola R F, Sala R, Bussolati O, et al. FEBS Lett, 2001, 490(1/2): 11 11172802 10.1016/s0014-5793(01)02126-3

[45] Amaya M L, Inguva A, Pei S, et al. Blood, 2022, 139(4): 584 34525179 10.1182/blood.2021013201 PMC8796651

[46] Singleton D C, Harris A L. Expert Opin Ther Targets, 2012, 16(12): 1189 23009153 10.1517/14728222.2012.728207

[47] Furusawa A, Miyamoto M, Takano M, et al. Carcinogenesis, 2018, 39(6): 758 29617730 10.1093/carcin/bgy033

[48] Yang L, Achreja A, Yeung T L, et al. Cell Metab, 2016, 24(5): 685 27829138 10.1016/j.cmet.2016.10.011 PMC7329194

